# Tetrathiomolybdate Decreases the Expression of Alkaline Phosphatase in Dermal Papilla Cells by Increasing Mitochondrial ROS Production

**DOI:** 10.3390/ijms24043123

**Published:** 2023-02-04

**Authors:** Fan Li, Hongli Liu, Xiaojing Wu, Zhicheng Song, Haojia Tang, Maohua Gong, Lei Liu, Fuchang Li

**Affiliations:** 1 Key Laboratory of Efficient Utilization of Non-Grain Feed Resources (Co-Construction by Ministry and Province), Ministry of Agriculture and Rural Affairs, Shandong Provincial Key Laboratory of Animal Biotechnology and Disease Control and Prevention, Department of Animal Science, Shandong Agricultural University, Taian 271018, China; 2 Hebei Key Laboratory of Specialty Animal Germplasm Resources Exploration and Innovation, Department of Animal Science and Technology, Hebei Normal University of Science and Technology, Qinhuangdao 066004, China

**Keywords:** dermal papilla cells, copper, ROS, hair follicle, ALP

## Abstract

Dermal papilla cells (DPCs) play important roles in hair growth regulation. However, strategies to regrow hair are lacking. Here, global proteomic profiling identified the tetrathiomolybdate (TM)-mediated inactivation of copper (Cu) depletion-dependent mitochondrial cytochrome c oxidase (COX) as the primary metabolic defect in DPCs, leading to decreased Adenosine Triphosphate (ATP) production, mitochondrial membrane potential depolarization, increased total cellular reactive oxygen species (ROS) levels, and reduced expression of the key marker of hair growth in DPCs. By using several known mitochondrial inhibitors, we found that excessive ROS production was responsible for the impairment of DPC function. We therefore subsequently showed that two ROS scavengers, N-acetyl cysteine (NAC) and ascorbic acid (AA), partially prevented the TM- and ROS-mediated inhibition of alkaline phosphatase (ALP). Overall, these findings established a direct link between Cu and the key marker of DPCs, whereby copper depletion strongly impaired the key marker of hair growth in the DPCs by increasing excessive ROS production.

## 1. Introduction

Copper (Cu) is a redox-active metal ion that regulates many important processes in aerobic organisms ranging from bacteria to mammals [1,2,3,4]. Copper has been largely studied in the context of enzyme cofactors, the unstable forms of which can trigger oxidative stress and free-radical damage [5]. Therefore, cellular homeostasis requires intracellular copper concentrations to be maintained at extremely low levels, thus preventing the accumulation of intracellular copper, which is harmful to cells [6,7,8]. However, recent research has demonstrated the regulatory roles of copper in diverse biological processes such as the cyclic-AMP-dependent lipolysis in 3T3-L1 adipocytes [9], the oxidation of cytochrome c [10], the regulation of carbon entry into the tricarboxylic acid (TCA) cycle [7], autophagy, and the pathogenesis of lung adenocarcinoma [11].

The dermal papilla is composed of highly specialized mesenchymal cells called dermal papilla cells (DPCs), which are located at the base of the hair follicle [12] and play a crucial role in the epithelial–mesenchymal interactions that regulate the morphogenesis and cycling of hair follicles [13]. It has been reported that DPCs are essential for the activity of hair follicle stem cells (HFSCs) [14]. The signals emanating from DPCs, such as fibroblast growth factor (FGF), Wingless/Integrated (Wnt), and bone morphogenetic proteins (BMP), are essential for the transition from the telogen phase to the anagen phase of the hair follicle cycle [15,16,17,18]. DPCs are intrinsically heterogeneous and can induce de novo hair follicle formation when transplanted to a different location on the skin. Importantly, the source of DPCs determines the type of newly formed hair [17,19]. Ohyama et al. reported that alkaline phosphatase (ALP) expression is a biological marker of DPCs [12,13]. Moreover, ALP activity is correlated with the hair-inducing capacity of DPCs [18,20].

In this context, we became interested in the relationship between copper and DPC function. Recent studies have highlighted the role of copper as an important regulator of cellular metabolism. As the catalytic core of cytochrome c oxidase (COX), copper is necessary for oxidative phosphorylation (OXPHOS) [21]. However, the hypoxic environment is more conducive to the special functions of DPCs; in particular, their ALP activity [22]. Previous studies have revealed the positive effect of copper on hair follicle development in mammals. In these studies, copper administration increased the number of secondary follicles and promoted hair growth [23,24,25]. In accordance, in the present study, we found that copper depletion led to excessive reactive oxygen species (ROS) production, which impaired DPC function. Moreover, we showed that the function of DPCs was not affected by the energy supply. Taken together, our results suggest a mechanistic link between mitochondrial metabolism and the capacity of DPCs to induce hair growth.

## 2. Results

### 2.1. Copper Depletion Impairs ALP Activity

**Figure 1:**
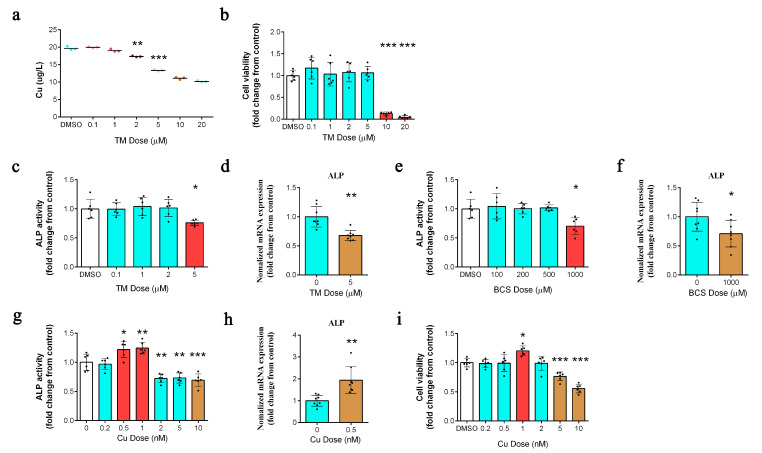
(**a**) Measurement of intracellular copper levels in dermal papilla cells (DPCs) after 72 h of treatment with different tetrathiomolybdate (TM) concentrations (*n* = 3/group). ** *p* < 0.01, *** *p* < 0.001. (**b**) A cell counting kit-8 (CCK-8) assay was used to assess the effect of different TM concentrations on DPCs viability after 72 h of treatment (*n* = 6/group); *** *p* < 0.001. (**c**) Measurement of ALP activity in DPCs after 72 h of treatment with TM (*n* = 6/group); * *p* < 0.05. (**d**) qPCR analysis of the transcript levels of the ALP gene in control and TM-treated (5 µM) DPCs after 72 h of treatment (*n* = 8/group); ** *p* < 0.01. (**e**) Measurement of ALP activity in DPCs after 72 h of treatment with BCS (*n* = 6/group); * *p* < 0.05. (**f**) qPCR analysis of the transcript levels of the ALP gene in control and BCS-treated (1000 µM) DPCs after 72 h of treatment (*n* = 8/group); * *p* < 0.05. (**g**) Measurement of ALP activity in DPCs after 72 h of treatment with copper (*n* = 6/group); * *p* < 0.05, ** *p* < 0.01, *** *p* < 0.001. (**h**) qPCR analysis of the transcript levels of the ALP gene in control and copper-treated (0.5 nM) DPCs after 72 h of treatment (*n* = 8/group); ** *p* < 0.01. (**i**) A cell counting kit-8 (CCK-8) assay was used to assess the effect of different copper concentrations on DPC viability after 72 h of treatment (*n* = 6/group); * *p* < 0.05, *** *p* < 0.001.

We treated DPCs with multiple concentrations of tetrathiomolybdate (TM) to identify the lowest dose that could effectively deplete intracellular copper levels. Seventy-two hours of TM (5 µM) treatment efficiently depleted intracellular copper levels (Figure 1a) without affecting cell viability (Figure 1b). TM-mediated copper depletion reduced ALP activity (Figure 1c) and *ALP* mRNA levels (Figure 1d). Similar results were obtained with another copper chelator, bathocuproinedisulfonic acid disodium salt (BCS) (1000 µM) (Figure 1e,f). Moreover, the ALP activity (Figure 1g) and *ALP* mRNA levels (Figure 1h) were significantly increased following the addition of copper (as 0.5 nM copper chloride, without serum) without affecting cell viability (Figure 1i).

### 2.2. Copper Depletion Reduces Mitochondrial Activity and Increases ROS Production

**Figure 2:**
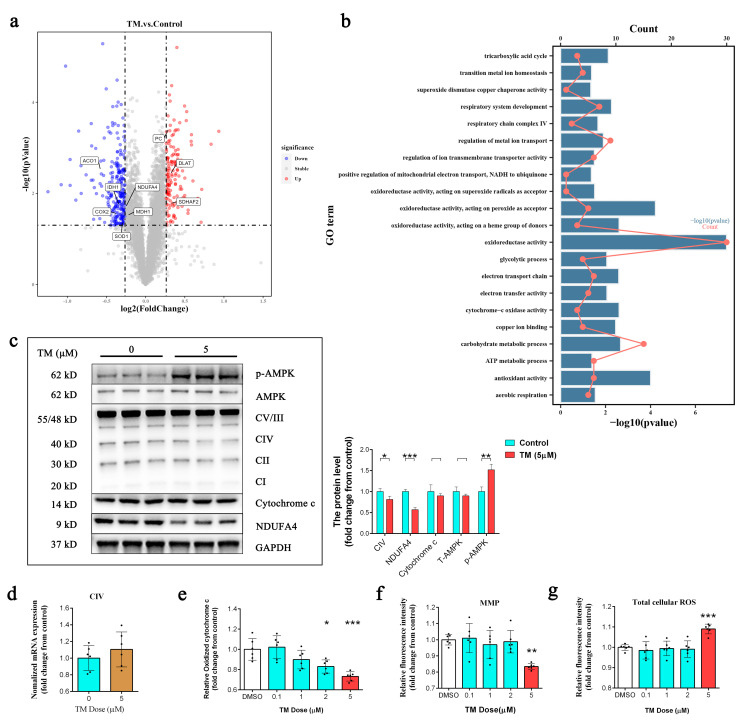
(**a**) Tandem mass tags (TMT) multiplexed quantitative proteomic analysis of control and TM (5 µM)-treated DPCs (*n* = 3). The red points represent significantly upregulated proteins, the blue points represent significantly downregulated proteins, and the gray points represent proteins showing no change. The *x*-axis represents the Log2(fold change), and the *y*-axis represents the −Log10(*p*-value). (**b**) Gene ontology (GO) analysis of downregulated proteins after 72 h TM (5 µM) treatment in DPCs. (**c**) Western blot analysis of the protein levels of MTCO1, NDUFA4, and cytochrome c after 72 h of TM (5 µM) treatment (*n* = 3). * *p* < 0.05; ** *p* < 0.01; *** *p* < 0.001. (**d**) qPCR analysis of the transcript levels of the CIV gene after 72 h of TM (5 µM) treatment (*n* = 6/group). (**e**) Cytochrome c oxidase activity measured after 72 h of treatment with TM (*n* = 6/group). * *p* < 0.05; *** *p* < 0.001. (**f**) Measurement of mitochondrial membrane potential (MMP) in DPCs after 72 h of TM treatment (*n* = 6/group); ** *p* < 0.01. (**g**) Measurement of total cellular ROS levels in DPCs after 72 h of TM (*n* = 6/group); *** *p* < 0.001.

DPCs were treated with TM for 72 h and total protein expression was quantified using liquid chromatography–mass spectrometry (LC–MS). Compared to the control group, the expression of 256 proteins was significantly reduced and the expression of 145 proteins was significantly increased in the TM-treated group (fold change < 0.83, *p* < 0.05) (Supplementary Tables S3 and S4). As expected, the expression of several subunits of mitochondrial cytochrome c oxidase (COX) and enzymes associated with central carbon metabolism was specifically reduced (Figure 2a). The gene ontology (GO) analysis of downregulated proteins showed the enrichment of pathways involved in the mitochondrial electron transport chain and cytochrome c oxidase activity (Figure 2b). Western blotting confirmed a reduction in the levels of COX subunits, including the mitochondrially encoded cytochrome c oxidase I (CIV) and NDUFA4 mitochondrial complex associated (NDUFA4) (Figure 2c), which was not accompanied by changes in mRNA levels (Figure 2d). As expected, this resulted in a significant reduction in the oxidation of COX (Figure 2e), without impacting the protein levels of cytochrome c (Figure 2c). Moreover, we observed that copper depletion increased the protein levels of AMP-activated protein kinase (AMPK) phosphorylation at Thr172 (Figure 2c), depolarized the mitochondrial membrane potential (MMP) (Figure 2f), and increased the total cellular ROS levels (Figure 2g).

### 2.3. Copper Depletion Reprograms Metabolism

**Figure 3:**
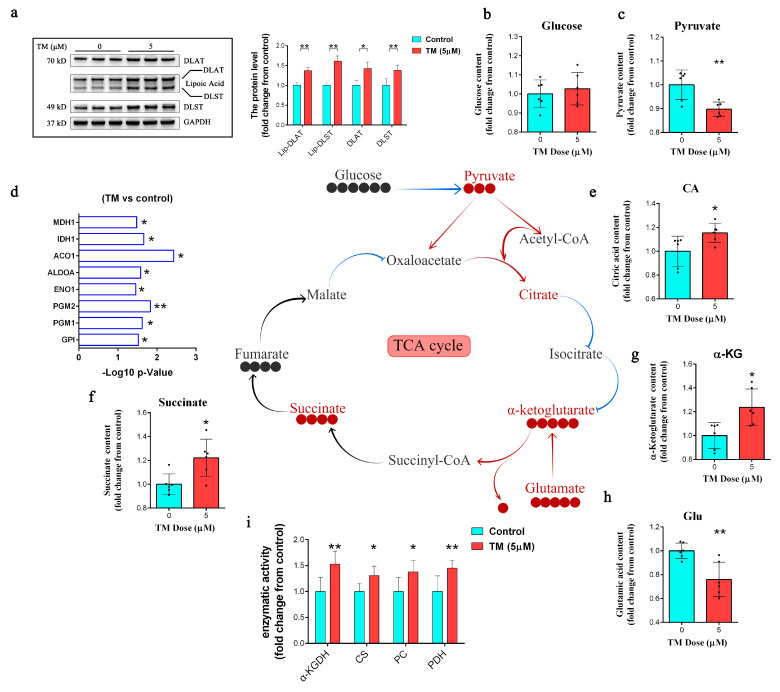
(**a**) Western blot analysis of the protein levels of DLAT, DLST, and lipoylated proteins after 72 h of TM (5 µM) treatment (*n* = 3); * *p* < 0.05; ** *p* < 0.01. (**b**,**c**) Glucose and pyruvate consumption analysis after 72 h of TM (5 µM) treatment in DPCs, and the supernatant was replaced 24 h before collection in both groups (*n* = 6); ** *p* < 0.01. (**d**) Downregulated protein analysis after 72 h of TM (5 µM) treatment in DPCs. The *x*-axis represents the -Log10(*p*-value) and the significance was calculated using multiple t-tests (*n* = 3/group); * *p* < 0.05, ** *p* < 0.01. (**e**–**h**) Measurement of the contents of citric acid, succinate, α-ketoglutarate, and glutamic acid after 72 h of treatments with TM (5 µM), and the supernatant was replaced 24 h before collection in both groups (*n* = 6/group); * *p* < 0.05; ** *p* < 0.01. (**i**) Measurement of pyruvate carboxylase (PC), pyruvate dehydrogenase (PDH), citrate synthase (CS), and α-ketoglutarate dehydrogenase (α-KGDH) activity in DPCs after 72 h of treatment with TM (5 µM), and the supernatant was replaced 24 h before collection in both groups (*n* = 6/group); * *p* < 0.05, ** *p* < 0.01.

Western blotting confirmed the increase in the levels of the mitochondrial lipoylated proteins dihydrolipoyl transacetylase (DLAT) and dihydrolipoamide succinyltransferase (DLST) after copper depletion with TM (Figure 3a). The nutrient analysis of DPC supernatants showed that treatment with TM had no significant effect on glucose consumption (Figure 3c) but significantly increased pyruvate consumption (Figure 3d). Moreover, the proteomic analysis showed that the TM-treated DPCs had lower levels of enzymes involved in glycolysis and the TCA cycle (Figure 3e). In addition, TM-mediated copper depletion led to an accumulation of citric acid, succinate, α-ketoglutarate, and glutamic acid (Figure 3f–i). We also observed the increased activities of pyruvate carboxylase (PC), pyruvate dehydrogenase (PDH), citrate synthase (CS), and α-ketoglutarate dehydrogenase (α-KGDH) (Figure 3j), all of which are enzymes that regulate carbon entry into the TCA cycle [7,26,27].

### 2.4. Copper Depletion Inhibits Mitochondrial ATP Production

**Figure 4:**
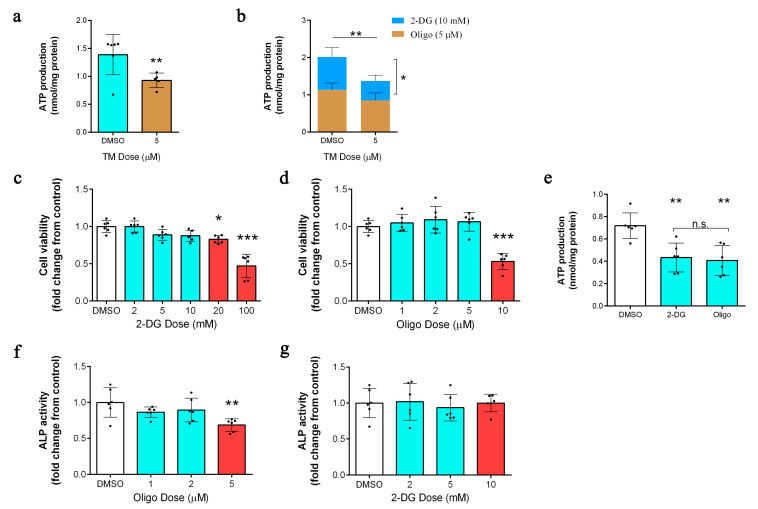
(**a**) ATP production after 72 h of treatment with TM (5 µM) (*n* = 6/group); ** *p* < 0.01. (**b**) After TM treatment for 48 h, measured ATP production after 24 h of treatments with 2-DG (10 mmol) and oligo (5 µM) (*n* = 6/group); * *p* < 0.05, ** *p* < 0.01. (**c**,**d**) A cell counting kit-8 (CCK-8) assay was used to assess the effect of 2-DG and oligo concentrations on DPC viability after 24 h of treatment (*n* = 6/group); * *p* < 0.05, *** *p* < 0.001. (**e**) ATP production after 24 h of treatments with 2-DG (10 mmol) and oligo (5 µM) (*n* = 6/group); ** *p* < 0.01. (**f**,**g**) Measurement of ALP activity in DPCs after 24 h of treatments with 2-DG and oligo (*n* = 6/group); ** *p* < 0.01.

TM-mediated copper depletion led to a significant reduction in ATP production (Figure 4a). Moreover, we used 2-deoxyglucose (2-DG) and oligomycin to show that TM treatment significantly inhibited mitochondrial ATP production rather than glycolysis without affecting cell viability (Figure 4b–d). We therefore speculated whether the reduction in ATP production as a result of copper depletion led to ALP inactivation. As expected, both 2-DG and oligomycin significantly reduced ATP production (Figure 4e). However, oligomycin treatment significantly inhibited ALP activity (Figure 4f), while 2-DG treatment had no significant effect on ALP activity (Figure 4g). Together, these results indicated that the decrease in ATP production could not be fully explained by the inhibition of ALP.

### 2.5. ROS Production Impairs ALP Activity

**Figure 5:**
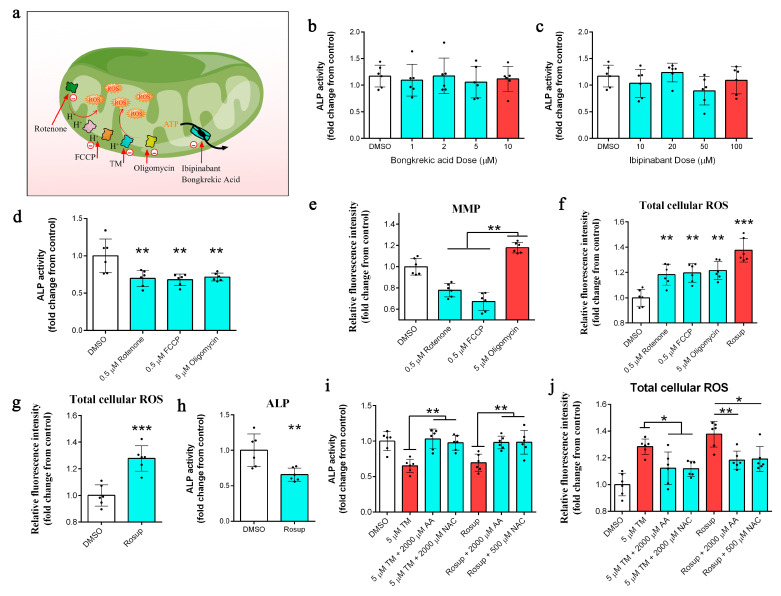
(**a**) Diagram depicting the targets of various mitochondrial perturbagens. (**b**,**c**) Measurement of ALP activity in DPCs after 24 h of treatments with bongkrekic acid and ibipinabant (*n* = 6/group). (**d**) Measurement of ALP activity in DPCs after 24 h of treatments with rotenone, FCCP, and oligomycin (*n* = 6/group); ** *p* < 0.01. (**e**) Measurement of mitochondrial membrane potential (MMP) in DPCs after 24 h of treatments with rotenone, FCCP, and oligomycin (*n* = 6/group); ** *p* < 0.01. (**f**) Measurement of total cellular ROS levels in DPCs after 24 h of treatments with rotenone, FCCP, and oligomycin (*n* = 6/group); ** *p* < 0.01, *** *p* < 0.001. (**g**) Measurement of total cellular ROS levels in DPCs after 24 h of treatments with Rosup (*n* = 6/group); *** *p* < 0.001. (**h**) Measurement of ALP activity in DPCs after 24 h of treatments with Rosup (*n* = 6/group); ** *p* < 0.01. (**i**) Measurement of ALP activity in DPCs after 48 h of treatments with TM, Rosup, AA, and NAC (*n* = 6/group); ** *p* < 0.01. (**j**) Measurement of total cellular ROS levels in DPCs after 48 h of treatments with TM, Rosup, AA, and NAC (*n* = 6/group); * *p* < 0.05, ** *p* < 0.01.

Neither of the adenine nucleotide translocator (ANT) inhibitors (bongkrekic acid and ibipinabant) affected ALP activity (Figure 5b,c) or cell viability (Figure S1a,b). We next showed that both rotenone and carbonyl cyanide 4-(trifluoromethoxy)phenylhydrazone (FCCP) reduced ALP activity (Figure 5c) as much as oligomycin, despite their opposing effect on the MMP (Figure 5d). Neither rotenone nor FCCP affected DPC viability at the doses used (Figure S1c,d). However, all the mitochondrial inhibitors induced the production of ROS (Figure 5e). We next confirmed that ROS was the main cause of ALP inhibition by using Rosup (a ROS inducer) (Figure 5f); Rosup did not affect cell viability (Figure S1e). Moreover, NAC and AA, two ROS scavengers, partially prevented the TM- and Rosup-mediated inhibition of ALP (Figure 5g) and reduced the generation of ROS (Figure 5h).

### 2.6. Metabolic Defects Mediated by Copper Depletion Are Reversible

**Figure 6:**
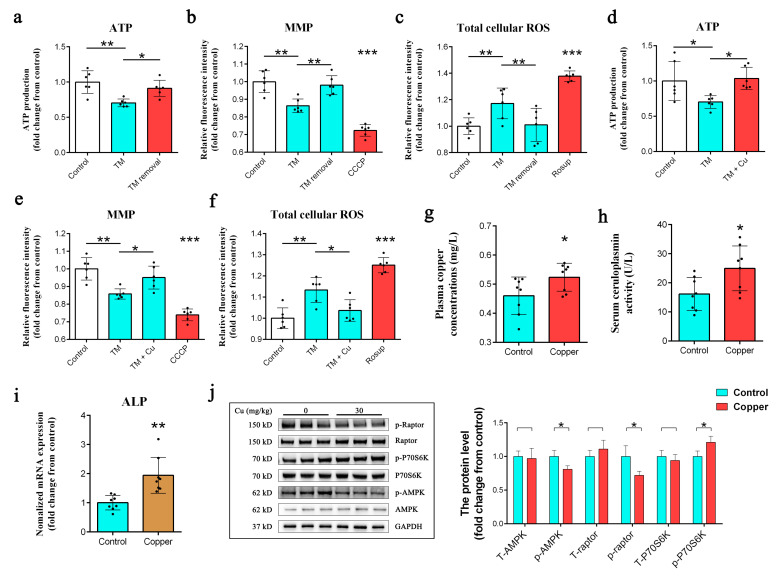
(**a**) Effect of TM removal (48 h of TM removal after 48 h of treatment with 5 µM TM) from treated DPCs on ATP production (*n* = 6/group); * *p* < 0.05, ** *p* < 0.01. (**b**) Effect of TM removal (48 h of TM removal after 48 h of treatment with 5 µM TM) from treated DPCs on MMP (*n* = 6/group); ** *p* < 0.01, *** *p* < 0.001. (**c**) Effect of TM removal (48 h of TM removal after 48 h of treatment with 5 µM TM) from treated DPCs on the total cellular ROS (*n* = 6/group); ** *p* < 0.01, *** *p* < 0.001. (**d**) Effect of copper addition (treated for 48 h with copper (CuCl_2_ (5 nM)) after 48 h of treatment with 5 µM TM) to treated DPCs on ATP production (*n* = 6/group); * *p* < 0.05. (**e**) Effect of copper addition (treated for 48 h with copper (CuCl_2_ (5 nM)) after 48 h of treatment with 5 µM TM) to treated DPCs on MMP (*n* = 6/group); * *p* < 0.05, ** *p* < 0.01, *** *p* < 0.001. (**f**) Effect of copper addition (treated for 48 h with copper (CuCl_2_ (5 nM)) after 48 h of treatment with 5 µM TM) to treated DPCs on the levels of total cellular ROS (*n* = 6/group); * *p* < 0.05, ** *p* < 0.01, *** *p* < 0.001. (**g**,**h**) Effect of copper supplementation (30 mg/kg) on the levels of plasma copper and serum ceruloplasmin (*n* = 8/group); * *p* < 0.05. (**i**) qPCR analysis of the transcript levels of the ALP gene in control and copper group dorsal skin from Rex rabbits (*n* = 8/group); ** *p* < 0.01. (**j**) Western blot showing the protein levels of AMPK, p-AMPK (Thr172), raptor, p-raptor (792), p70S6K, and p-P70S6K (Thr389) in the dorsal skin of Rex rabbits fed copper (*n* = 3/group); * *p* < 0.05.

We next replaced the culture medium of the TM-treated DPCs with a TM-free medium and found that within 48 h of TM removal, ATP production was restored to normal levels (Figure 6a). Moreover, TM removal also restored the MMP (Figure 6b) and decreased total cellular ROS levels (Figure 6c). Additionally, ATP production was also rescued by Cu (5 nM) add-back to the TM-treated DPCs (Figure 6d) without affecting cell viability (Figure S1f). As expected, the addition of copper also increased the MMP (Figure 6e) and reduced the cellular ROS levels (Figure 6f). Furthermore, when 3-month-old Rex rabbits were fed a copper diet (39.1 mg/kg) for 5 weeks, their plasma copper and serum ceruloplasmin levels increased (Figure 6g). The corresponding increase in *ALP* mRNA levels (determined using quantitative real-time (qRT)-PCR) was also observed in the dorsal skin of these Rex rabbits (Figure 6h). Moreover, we found that the phosphorylation of AMPK (at Thr172) and raptor (at Ser792) decreased, while the phosphorylation of the mammalian target of rapamycin complex I (mTORC1) downstream target P70S6K (at Thr389) increased (Figure 6i).

## 3. Discussion

Numerous studies have shown that dynamic changes in intracellular copper concentration can regulate protein function, cell fate, and cell health [7,28]. Our results showed that one of the mechanisms by which copper depletion alters the function of DPCs is the inhibition of COX, a key component of the mitochondrial electron transport chain (ETC), leading to excessive ROS production. We also showed that copper chelation by TM [10] effectively depleted intracellular copper without impacting DPC viability. Copper depletion, in turn, inhibited ALP and decreased its expression. ALP is a classical biological marker of DPCs, and its activity is positively correlated with the capacity of DPCs to induce hair follicle growth [18,20]. The source of copper in the cell culture medium is serum [7]. By contrast, the ALP activity and *ALP* mRNA levels were increased by the addition of copper without serum. Taken together, these results highlight the mechanistic link between copper and the key marker of DPCs.

Global proteomic profiling identified the TM-mediated inactivation of copper-dependent mitochondrial COX as the primary metabolic defect in DPCs by showing that the expression of multiple catalytic core subunits of COX was significantly reduced. It has been reported that in the absence of copper atoms, the COX holoenzyme cannot undergo assembly and the catalytic core subunits are rapidly degraded [10,21]. Subsequently, Western blotting confirmed the reduction in the protein levels of the COX subunits CIV and NDUFA4 without affecting the mRNA levels of COX1. We surmised that the TM-mediated reduction in the levels of COX subunits was associated with the inhibition of cytochrome c oxidation. As the terminal metalloenzyme of the mitochondrial ETC, COX is involved in the generation of the MMP. Thus, the inhibition of COX usually leads to MMP uncoupling and excessive ROS production [29,30]. As expected, copper depletion significantly decreased the MMP and increased the total cellular ROS levels. In brief, the TM-induced mitochondrial changes likely reprogram cellular metabolism.

Tsvetkov et al. reported that excessive intracellular copper binding to lipoylated proteins (e.g., DLAT and DLST) results in lipoylated protein aggregation and the loss of enzymatic function [7,26,27]. We observed that copper depletion increased the activities of the lipoylated TCA enzymes PDH and α-KGDHC, which was associated with increased protein lipoylation. The OXPHOS and the TCA cycle are coupled. Therefore, the continuous transfer of electrons in the ETC is essential for maintaining the function and flux of the TCA cycle [7,31]. The results of the proteomics analysis showed that the expression of multiple enzymes involved in glycolysis and the TCA cycle (in particular, those involved in the conversion of glucose to pyruvate) was significantly reduced following the TM treatment of DPCs. Nutrient analysis in DPCs showed that the accumulation of citric acid (CA), α-ketoglutarate (α-KG), and succinate may be due to the inhibition of the TCA cycle following OXPHOS disruption. The term anaplerosis describes the multiple inputs into the TCA cycle [31]. Two important anaplerotic mechanisms are (1) the conversion of pyruvate to mitochondrial oxaloacetic acid (OAA) by pyruvate carboxylase; and (2) the activation of glutaminolysis, which converts glutamine to α-KG [31]. We observed that the consumption of pyruvate increased, and the intracellular glutamate levels decreased after the TM treatment of DPCs. This is indicative of increased anaplerosis.

Thus, TM-mediated copper depletion reprogrammed cell metabolism and increased the input into the TCA cycle. However, the generation of energy by glycolysis and the TCA cycle is inefficient compared to OXPHOS [32]. Therefore, by using 2-DG (a glycolysis inhibitor) [7] and oligomycin (an ATP synthase inhibitor) [10], we confirmed that TM-mediated copper depletion significantly inhibited mitochondrial ATP production rather than glycolysis. The differentiation and activation of stem cells are usually accompanied by changes in carbohydrate metabolism, characterized by a transition from low-energy-supplying (glycolytic metabolism) to high-energy-supplying (OXPHOS) pathways [28,32]. Therefore, we wondered whether the TM-mediated decrease in ATP production led to the loss of the hair-inducing capacity of DPCs. Unexpectedly, oligomycin, but not 2-DG treatment, significantly inhibited ALP activity. Ibrahim et al. demonstrated the unique physiological function of mitochondrial ATP production, whereby ATP production is coupled with the uptake and transport of fatty acids [33]. We therefore used bongkrekic acid and ibipinabant, two structurally different ANT inhibitors [33] that block mitochondrial ATP transport from the matrix to the cytoplasm, in further experiments. However, neither of the ANT inhibitors affected ALP activity, which indicated that there was no direct link between ATP production and the hair-inducing capacity of DPCs. We next tested several known mitochondrial inhibitors to determine if the perturbation of mitochondrial function impacted the activity of ALP. Both rotenone and CCCP, which inhibit complex I [34] and the MMP [35], respectively, reduced ALP activity to the same extent as oligomycin, despite their opposing effects on the MMP. These results showed that the inactivation of ALP did not directly depend on the MMP, although MMP is essential for the maintenance of cellular health and viability [36]. One thing that all of the mitochondrial inhibitors did have in common was their induction of mitochondrial ROS production [34,35,36]. ROS are the normal byproduct of aerobic metabolism and are mainly produced by mitochondria [37]. Recent studies have demonstrated that ROS act as an essential second messenger to mediate different intracellular processes [38,39,40,41,42]. However, excessive ROS accumulation leads to oxidative stress and can severely compromise the capacity of DPCs to induce and support hair growth [22,29]. While complex I and complex II are the direct sites of ROS generation, COX activity perturbations can indirectly increase mitochondrial ROS production [43], which is consistent with our results. We next used Rosup, a ROS inducer, to confirm that increased ROS levels were the main cause of ALP inactivation. Moreover, NAC and AA, two ROS scavengers [40,44,45], partially prevented the TM- and Rosup-mediated inhibition of ALP and reduced the generation of cellular ROS. We were then interested in determining whether TM-mediated copper depletion had induced a permanent metabolic change in DPCs. We therefore replaced the medium of TM-treated DPCs with TM-free supernatant for 48 h and found that ATP production in these cells had reverted to normal levels. Moreover, we observed that the removal of TM increased the MMP and decreased the total cellular ROS levels. Similar results were obtained by adding copper to TM-treated DPCs. These findings were consistent with those of Ramchandani et al. [10]. Previous studies have revealed the positive effects of copper on hair follicle development in mammals, including increases in the number of secondary follicles and improved hair growth [23,24,25]. In accordance with previous research [24], we showed that the copper supplementation of diets fed to Rex rabbits significantly increased their plasma copper levels and serum ceruloplasmin activity. Additionally, we observed an increase in the *ALP* mRNA levels. Previous research has reported that the inhibition of mTOR signaling by rapamycin resulted in delayed hair cycle initiation (in the first phase of the cycle). Moreover, AMPK inhibition activates the mTORC1 pathways [46,47]. Here, we observed that dietary copper supplementation reduced AMPK phosphorylation in the dermis of Rex rabbits. Consistent with previous research, the addition of copper also decreased the phosphorylation of raptor and increased the phosphorylation of mTORC1 downstream target P70S6K in the dermis of Rex rabbits [10]. It has been reported that mTORC1 signaling plays a critical role in the regulation of stem cell activation [47]. Thus, the activation of mTORC1 may be the result of the increased mRNA levels of ALP observed after copper supplementation.

## 4. Materials and Methods

### 4.1. Materials

Bathocuproinedisulfonic acid (BCS) (B1125), tetrathiomolybdate (TM) (323446), ascorbic acid (AA) (A92902), N-acetyl-*_L_*-cysteine (NAC) (A7250), carbonyl cyanide m-chlorophenylhydrazone (CCCP) (C2920), and rotenone (R8875) were purchased from Sigma Aldrich (Sigma, St. Louis, OH, USA). Rosup (S0033S), 2-deoxyglucose (2-DG) (ST1024), and oligomycin A (SC0366) were purchased from Beyotime (Beyotime, Shanghai, China). Bongkrekic acid was purchased from Shanghai Topscience Technology Co., Ltd., (T36067). Ibipinabank was purchased from Shanghai Aladdin Technology Co., Ltd., (S287862). The graphical abstract and Figure 5a were drawn by Figdraw (www.figdraw.com, accessed on 6 January 2022).

### 4.2. Animals

Eighty 90-day-old Rex rabbits (supplied by the Taishan Rabbit factory, Taian, China) with an average body weight of 1914.125 g were randomly divided into two groups, with 40 replicates (20 males, 20 females) in each group. The Rex rabbits were housed in single cages with free access to food and water. Natural lighting and ventilation were maintained throughout the experimental period and the temperature of the rabbit room was controlled at 20–25 °C. The composition and chemical analysis of the basal diet are shown in Supplementary Table S1. The treatments were a basal diet (measured Cu content 8.4 mg/kg) or a basal diet supplemented with 30 mg/kg Cu (measured Cu content 39.1 mg/kg). The trial lasted for five weeks. At the end of the trial, 8 rabbits (half male and half female) per group were electrically stunned (70 V, pulsed direct current, 50 Hz for 5 s) and every effort was made to minimize discomfort and suffering.

### 4.3. Cells

Dermal papilla cells (DPCs) from Rex rabbits were kindly provided by Professor Xin Sheng Wu (College of Animal Science and Technology, Yangzhou University, Jiangsu, China) and were identified as previously described. The results showed that the isolated DPCs had high alkaline phosphatase activity and the marker proteins α smooth muscle actin (α-SMA) and versican (Vim) were positive [48].

### 4.4. Cell Culture

DPCs were cultured at 37 °C in an incubator with 5% CO_2_. Before each experiment, DPCs were serum-starved overnight in Dulbecco’s modified Eagle’s medium (DMEM; Thermo Fisher, Carlsbad, CA, USA) without fetal bovine serum (FBS).

### 4.5. Measurement of ATP Content

ATP content was determined using an ATP detection kit (S0026, Beyotime, Shanghai, China). Fluorescence was measured with a fluorescence microplate reader (BioTek Instruments, Winooski, VT, USA). The ATP content was normalized to the protein content.

### 4.6. Cytochrome C Oxidase Activity

COX activity was measured using a Mitochondrial Respiratory Chain Complex IV Activity Kit (BC0945; Beijing Solarbio Science & Technology Co., Ltd., Beijing, China). The difference in activity between the control and bathocuproinedisulfonic acid (BCS) treatment groups was determined at 550 nm with a Varioskan LUX microplate reader (Thermo Fisher) using the 1st- and 15th-minute readings. Complex IV activity was normalized to the protein content.

### 4.7. Assay for Mitochondrial Membrane Potential (MMP)

The MMP was determined with an MMP detection kit (C2006, Beyotime, Shanghai, China). The chemiluminescence signal was detected using a fluorescence microplate reader (BioTek Instruments). CCCP (Carbonyl cyanide m-chlorophenylhydrazone) (a membrane uncoupling chemical) was used as a positive control. The results were presented as relative fluorescence intensity and were normalized to that of the control group.

### 4.8. Determination of Total Cellular ROS Concentrations

Total cellular ROS levels were measured using a fluorescent molecular probe (20,70-dichlorodihydrofluorescein diacetate (DCFH-DA); S0033S, Beyotime, Shanghai, China). Rosup, a ROS inducer, served as a positive control. The results were presented as fluorescence intensity and were normalized to that of the control group.

### 4.9. Elisa Assay

The enzyme activity of α-KGDH, CS, PC, and PDH was detected according to the instructions of the assay kit (BC0715, BC1060, BC0730, BC0385, Solarbio, Beijing, China). The enzyme activity was normalized to the protein content.

### 4.10. ALP Activity

ALP activity was measured using an ALP activity kit (BC2140; Beijing Solarbio Science & Technology Co., Ltd., Beijing, China). The absorbance was detected at 510 nm and the activity of ALP was normalized to the protein content.

### 4.11. Determination of Metabolite Concentrations

The levels of glucose, pyruvate, citric acid, and glutamic acid were determined with an assay kit (BC2505, BC2200, BC2150, BC1580, Solarbio, Beijing, China). The levels of succinate and α-ketoglutarate were determined with an assay kit (ab204718, ab83431, abcam, Cambridge, UK).

### 4.12. Measurement of Copper Contents

TM-treated DPCs (2 × 10^7^) were digested with 50% HNO_3_ + 0.01% digitonin at 65 °C and the copper content was measured against copper standards using Analytik Jena novAA 400P (Jena, Germany).

### 4.13. RNA Isolation and Analysis

Total RNA extraction and quantitative reverse transcription polymerase chain reaction (qRT-PCR) were conducted as described previously [29,49,50,51]. Reverse transcription reactions (20 μL) contained 1000 ng of total RNA and 4 μL 5 × Evo M-MLVRT Master Mix (supplied by the Accurate Biotechnology Co., Ltd., Hunan, China). Real-time PCR analysis was carried out with an Applied Biosystems 7500 real-time PCR system (Applied Biosystems, Foster, CA, USA). Each RT reaction served as a template in a 20 μL PCR containing 0.2 mol/L of each primer and AceQ^®^ qPCR SYBR Green Master Mix (High ROX Premixed) (Q141-02, Vazyme Biotech Co.,Ltd. Nanjing, China). The gene for normalization was GAPDH (glyceraldehyde-3-phosphate dehydrogenase), and the results of relative mRNA quantification were verified using β-actin levels. The mRNA expression was analyzed using the 2^−ΔΔCT^ method [29,49,50,51]. Primer sequences are shown in Supplementary Table S2.

### 4.14. Western Blot

Total protein was extracted using a radioimmunoprecipitation assay (RIPA) lysis buffer (R0010, Solarbio, Beijing, China) with the addition of PMSF and protease inhibitor The proteins were separated on a 7.5–10% SDS polyacrylamide gel electrophoresis, transferred onto PVDF membranes at 200 mA at 4 °C (WB52002, Transfer buffer was supplied by New Cell Molecular Biotechnology Co., Ltd., Suzhou, China), and enclosed in closing solution (P30500, New Cell Molecular Biotechnology Co., Ltd., Shanghai, China) for 120 min. Protein detection was performed using enhanced chemiluminescence detection reagents (P0018FM, Beyotime, Shanghai, China). GAPDH (glyceraldehyde-3-phosphate dehydrogenase) was used as a loading control to quantify other protein levels. Western blots were developed and quantified with a BioSpectrum 810 Imaging System using VisionWorksLS 7.1 software (UVP LLC, Upland, CA, USA). The standard markers for protein molecular masses were supplied by Thermo (Cat# 26617, Thermo Fisher, Carlsbad, CA, USA). The membranes were probed with the required antibodies: OXPHOS antibody cocktail (Cat# ab110413, abcam), lipoic acid (Cat# ab58724, abcam), DLAT (Cat# 13426-1-AP, Proteintech), DLST (Cat# A13297, ABclonal), GAPDH (Cat# ab9485, abcam), AMPK (Cat# 10929-2-AP, Proteintech), p-AMPK (thr172) (Cat#50081, CST), P70S6K (Cat# 14485-1-AP, Proteintech), raptor (Cat# 20984-1-AP, Proteintech), p-P70S6K (Cat# 9204S, CST), and p-raptor (Cat# AP0928, ABclonal). The horseradish peroxidase (HRP)-conjugated goat anti-rabbit IgG (A0408, Beyotime, Shanghai, China) and goat anti-mouse IgG antibody (A0412, Beyotime, Shanghai, China) were supplied by Beyotime.

### 4.15. LC–MS Proteomic Analysis

Liquid chromatography–mass spectrometry (LC–MS) proteomic analysis was conducted by Shanghai iProteome Technology Co., Ltd. (Shanghai, China). Raw data files were processed using Firmiana. Gene ontology (GO) analysis was performed based on gene ontology (GO), the National Center for Biotechnology Information (NCBI), and the Uniprot database.

### 4.16. Statistical Analysis

The data were presented as means ± SD. Before analysis, all data were examined for homogeneity and normal distribution plots of variances among the treatments by using the UNIVARIATE procedure. Data from more than two groups were analyzed by analysis of variance (ANOVA), followed by Tukey’s HSD or Dunnett’s multiple comparisons. Data from two groups were analyzed by Student’s t-test. All statistical analyses were performed using SAS statistical software (SAS version 8.1, Cary, NC, USA). Differences were considered significant at *p* < 0.05.

## 5. Conclusions

The present study revealed an essential role for copper in regulating the proliferative capacity and functional features of DPCs. TM-mediated copper depletion severely compromised the key marker of hair growth in DPCs by causing mitochondrial dysfunction and increasing ROS production. Moreover, we showed that dietary copper supplementation significantly increased the mRNA levels of *ALPL* in rabbits. In summary, this work identifies a direct link between copper and the key marker of hair growth in DPCs.

## Data Availability

The datasets generated during and/or analyzed during the current study are available from the corresponding author upon reasonable request.

## Supplementary Materials

The following supporting information can be downloaded at: https://www.mdpi.com/article/10.3390/ijms24043123/s1.
