# Supplementary material for: Tetrathiomolybdate Decreases the Expression of Alkaline Phosphatase in Dermal Papilla Cells by Increasing Mitochondrial ROS Production
Source: Int J Mol Sci. 2023 Feb 4;24(4):3123. doi: 10.3390/ijms24043123 (PMC9960908; doi:10.3390/ijms24043123)
Supplement: Supplementary file 1 [file ijms-24-03123-s001.zip › ijms-2176619-supplementary.pdf]

Supplementary Figure S1

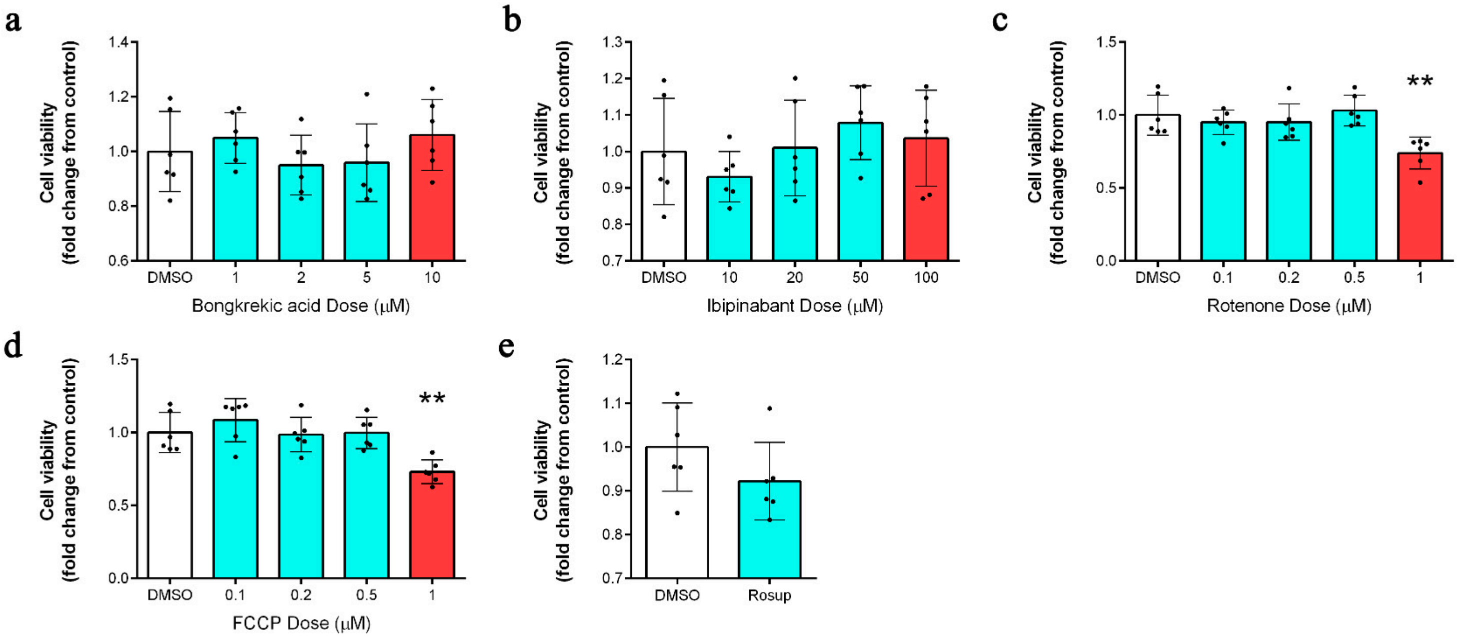

Supplementary Figure S1. **(a)** A cell counting kit-8 (CCK-8) assay was used to assess the effect of different Bongkrekic acid concentrations on DPCs viability after 24 h of treatment (n=6/group). **(b)** A cell counting kit-8 (CCK-8) assay was used to assess the effect of different Ibipinabant concentrations on DPCs viability after 24 h of treatment (n=6/group). **(c)** A cell counting kit-8 (CCK-8) assay was used to assess the effect of different rotenone concentrations on DPCs viability after 24 h of treatment (n=6/group); **p<0.01. **(d)** A cell counting kit-8 (CCK-8) assay was used to assess the effect of different FCCP concentrations on DPCs viability after 24 h of treatment (n=6/group); **p<0.01. **(e)** A cell counting kit-8 (CCK-8) assay was used to assess the effect of Rosup on DPCs viability after 24 h of treatment (n=6/group).

Supplementary Table S1. Composition and nutrient levels of the basal diet (air-dry basis) fed to Rex rabbits.

**Ingredients**	**%**	**Chemical analysis[2]**	**%**
Corn	10.50	DE (MJ/kg)	10.37
Soybean meal	6.00	DM	89.16
Corn germ meal	20.00	CP	16.87
Wheat bran	18.00	Ash	6.83
Husk powder	11.00	EE	4.39
Sunflower meal	12.00	CF	15.79
Alfalfa	6.00	Ca	0.70
Soya bean stem meal	12.00	P	0.54
Artemisia apiacea flour	3.00	Lys	0.53
Premix[1]	1.50	Met	0.89
Total	100.00		

[1] The premix provided the following per kilogram of diet: vitamin A, 10,000 IU; vitamin D4, 100 IU; vitamin E, 60 mg; vitamin K3, 2 mg; vitamin B1, 5 mg; vitamin B2, 10 mg, vitamin B11, 2.5 mg; vitamin B12, 0.01 mg; choline chloride, 600 mg; iron (as ferrous sulfate), 50 mg; zinc, 50 mg; selenium, 4 mg; iodine, 0.6 mg; manganese, 4 mg; $CaHPO_4$, 1,600 mg; NaCl, 4,800 mg; lysine, 1,000 mg; methionine, 2,000 mg; stone powder, 1,600 mg.

[2] Digestive energy is theoretically calculated, and other nutritional indicators are measured values.

Supplementary Table S2. Primer sequences for qPCR

**Genes 1)**	**GenBank accession number**	**Primer sequences (5′ - 3′)**	**Product size/bp**
CIV	NP_007551.1	F: 5′- CTATTTGGAGCTTGAGCTGGGATGG -3′<br>R:5′- AAGGCATGTGCGGTGACGATTAC -3′	128
ALPL	XM_017346489.1	F: 5′-ACAAGAAACCCTTCACTGCCATCC-3′<br>R:5′- GCCTGGTAGTTGTTGTGAGCGTAG -3′	112
GAPDH	NM_001082253.1	F: 5′-CACCAGGGCTGCTTTTAACTCT-3′<br>R:5′- CTTCCCGTTCTCAGCCTTGACC -3′	163
β-actin	XM_002722894.3	F: 5′- CGCAGAAACGAGACGAGATT -3′<br>R:5′- GCAGAACTTTGGGGACTTTG -3′	123

CIV = cytochrome c oxidase subunit 1; ALPL = alkaline phosphatase; GAPDH = glyceraldehyde phosphate dehydrogenase; β-actin = actin alpha.

Supplementary Table S3

Proteins down-regulated in dermal papilla cells (DPCs), with 72 hours of TM (5 μM) treatment.

protein	Description	FC	pValue	FDR	log2FC
COX2	cytochrome c oxidase subunit II	0.799161	0.029358	0.104596	-0.32344
ALDH1A1	aldehyde dehydrogenase 1 family member A1	0.746725	0.003475	0.05435	-0.42135
ITGA6	integrin subunit alpha 6	0.80012	0.001418	0.050986	-0.32171
ANXA8	annexin A8	0.78731	0.005129	0.058161	-0.345
GPI	glucose-6-phosphate isomerase	0.787076	0.029863	0.105064	-0.34543
DBI	diazepam binding inhibitor, acyl-CoA binding protein	0.767305	0.03783	0.114633	-0.38213
S100A11	S100 calcium binding protein A11	0.798322	0.036046	0.11162	-0.32496
ARNT	aryl hydrocarbon receptor nuclear translocator	0.70863	0.012193	0.077666	-0.4969
COL1A2	collagen type I alpha 2 chain	0.539113	0.014055	0.080384	-0.89134
FKBP3	FKBP prolyl isomerase 3	0.71723	0.015825	0.083209	-0.47949
ALDOA	aldolase, fructose-bisphosphate A	0.822101	0.026056	0.099048	-0.28261
GSTM2	glutathione S-transferase mu 2 (muscle)	0.654535	0.029226	0.10427	-0.61146
LDHB	lactate dehydrogenase B	0.781705	0.019435	0.088268	-0.3553
LDHA	lactate dehydrogenase A	0.804812	0.017852	0.086183	-0.31328
CRYL1	crystallin lambda 1	0.808318	0.006075	0.063602	-0.307
ACO1	aconitase 1	0.67551	0.003741	0.054832	-0.56595
PGM1	phosphoglucomutase 1	0.830079	0.02381	0.095783	-0.26868
ANPEP	alanyl aminopeptidase, membrane	0.785948	0.002822	0.053261	-0.34749
COL12A1	collagen type XII alpha 1 chain	0.512607	0.006677	0.064844	-0.96407
COL3A1	collagen type III alpha 1 chain	0.421227	0.009205	0.07061	-1.24733
GPX1	glutathione peroxidase 1	0.77305	0.002462	0.052154	-0.37137
CACNB2	calcium voltage-gated channel auxiliary subunit beta 2	0.824818	0.037755	0.114475	-0.27785
DAG1	dystroglycan 1	0.693198	0.00061	0.046446	-0.52866
ALCAM	activated leukocyte cell adhesion molecule	0.791281	0.044853	0.1233	-0.33774
SOD1	superoxide dismutase 1	0.817081	0.03403	0.109134	-0.29145
SPARC	secreted protein acidic and cysteine rich	0.590512	0.003916	0.055136	-0.75996
LAMB2	laminin subunit beta 2	0.806442	0.005402	0.059561	-0.31036
LRP1	LDL receptor related protein 1	0.734605	0.000477	0.042763	-0.44496
PRKDC	protein kinase, DNA-activated, catalytic subunit	0.825677	0.002892	0.053785	-0.27635
FCGRT	Fc gamma receptor and transporter	0.798561	0.000852	0.047849	-0.32453
IL18	interleukin 18	0.782299	0.021577	0.092514	-0.35421
FTO	FTO alpha-ketoglutarate dependent dioxygenase	0.80862	0.019667	0.088373	-0.30647
COL5A1	collagen alpha-1(V) chain	0.668521	0.006127	0.063744	-0.58096
IDH1	isocitrate dehydrogenase (NADP(+)) 1	0.80018	0.021762	0.092574	-0.3216
GET1	guided entry of tail-anchored proteins factor 1	0.801261	0.002476	0.052154	-0.31966
MAP1A	microtubule associated protein 1A	0.822047	0.008241	0.0678	-0.28271
CPNE1	copine I	0.822296	0.010052	0.072983	-0.28227
ANXA1	annexin A1	0.823154	0.022388	0.093892	-0.28077
GSN	gelsolin	0.783591	0.0154	0.082475	-0.35183
GLOD4	glyoxalase domain containing 4	0.758499	0.041704	0.120444	-0.39878
PLPP3	phospholipid phosphatase 3	0.725626	0.005733	0.061767	-0.4627

MIF	macrophage migration inhibitory factor	0.769684	0.026141	0.099088	-0.37766
GNPDA2	glucosamine-6-phosphate deaminase 2	0.750292	0.02226	0.093832	-0.41448
SARS1	seryl-tRNA synthetase 1	0.772002	0.012723	0.078033	-0.37332
STMN1	stathmin 1	0.779887	0.014768	0.08192	-0.35866
HDGF	heparin binding growth factor	0.8226	0.038508	0.115999	-0.28174
DPYSL3	dihydropyrimidinase like 3	0.767826	0.035399	0.110951	-0.38115
PCNA	proliferating cell nuclear antigen	0.803969	0.011021	0.075307	-0.31479
CTHRC1	collagen triple helix repeat containing 1	0.620308	0.000909	0.048318	-0.68894
PLD1	phospholipase D1	0.747525	0.000325	0.041472	-0.41981
MAB21L1	mab-21 like 1	0.772526	0.004615	0.057495	-0.37234
ATXN10	ataxin 10	0.778008	0.008867	0.069478	-0.36214
GULP1	GULP PTB domain containing engulfment adaptor 1	0.778601	0.00122	0.050296	-0.36104
PTPRK	protein tyrosine phosphatase receptor type K	0.821494	0.003626	0.05435	-0.28368
CMPK1	cytidine/uridine monophosphate kinase 1	0.82014	0.034781	0.110029	-0.28606
PGM3	phosphoglucomutase 3	0.779591	0.032229	0.107703	-0.35921
TGFB1I1	transforming growth factor beta 1 induced transcript 1	0.796945	0.004978	0.058161	-0.32745
COL5A2	collagen type V alpha 2 chain	0.627781	0.005107	0.058161	-0.67167
NFIB	nuclear factor I B	0.827597	0.006664	0.064844	-0.273
SEPTIN6	septin 6	0.798022	0.003257	0.053785	-0.3255
ARRB2	arrestin beta 2	0.812689	0.036755	0.113105	-0.29923
NCOA1	nuclear receptor coactivator 1	0.738626	0.002611	0.052654	-0.43708
MDH1	malate dehydrogenase 1	0.828711	0.033014	0.1084	-0.27106
PIBF1	progesterone immunomodulatory binding factor 1	0.713796	0.006441	0.064546	-0.48642
MYL10	myosin light chain 10	0.768868	0.046897	0.125749	-0.37919
CGNL1	cingulin like 1	0.833181	0.001665	0.051413	-0.2633
ARL3	ADP ribosylation factor like GTPase 3	0.825122	0.03611	0.111674	-0.27732
DNAJC8	DnaJ heat shock protein family (Hsp40) member C8	0.804211	0.003489	0.05435	-0.31435
FAM107B	family with sequence similarity 107 member B	0.752848	0.015375	0.082475	-0.40957
CAB39	calcium binding protein 39	0.746217	0.004257	0.056057	-0.42233
COLEC12	collectin subfamily member 12	0.776725	0.002267	0.052154	-0.36452
KIAA0754	KIAA0754 ortholog	0.804269	0.005743	0.061767	-0.31425
PYM1	PYM homolog 1, exon junction complex associated factor	0.817081	0.011032	0.075307	-0.29145
COBLL1	cordon-bleu WH2 repeat protein like 1	0.819836	0.004799	0.057689	-0.28659
GRB2	growth factor receptor bound protein 2	0.812141	0.010841	0.075051	-0.3002
MTPN	myotrophin	0.813539	0.04529	0.123603	-0.29772
ITGA1	integrin subunit alpha 1	0.784121	9.94E-05	0.037192	-0.35085
PDGFRB	platelet derived growth factor receptor beta	0.683782	5.20E-06	0.01604	-0.54839
LOC100342906	prothymosin alpha	0.762926	0.048763	0.128352	-0.39039
PDXK	pyridoxal kinase	0.786246	0.003477	0.05435	-0.34695
UCHL3	ubiquitin C-terminal hydrolase L3	0.817631	0.032563	0.108175	-0.29048
FSTL1	follistatin like 1	0.811594	0.030385	0.105064	-0.30117
LOC100343223	alcohol dehydrogenase [NADP(+)]	0.795033	0.045193	0.123577	-0.33091
TCF4	transcription factor 4	0.705514	5.09E-05	0.037192	-0.50325

MYL9	myosin light chain 9	0.734104	0.007416	0.066005	-0.44594
PGER5	prostaglandin-E(2) 9-reductase-like	0.766264	0.019212	0.088028	-0.38409
SPAG7	sperm associated antigen 7	0.807773	0.003209	0.053785	-0.30798
RNASEL	ribonuclease L	0.832315	0.003154	0.053785	-0.2648
DOCK5	dedicator of cytokinesis 5	0.764187	0.008706	0.068958	-0.388
MCEE	methylmalonyl-CoA epimerase	0.80072	0.004797	0.057689	-0.32063
COL6A1	collagen type VI alpha 1 chain	0.727613	0.0077	0.066901	-0.45876
LOC100344007	translationally-controlled tumor protein	0.806141	0.02272	0.094062	-0.3109
PPP1R21	protein phosphatase 1 regulatory subunit 21	0.826484	0.000321	0.041472	-0.27494
PRDX6	peroxiredoxin 6	0.734605	0.029919	0.105064	-0.44496
STAT2	signal transducer and activator of transcription 2	0.646817	0.010115	0.072983	-0.62857
SPATS2L	spermatogenesis associated serine rich 2 like	0.784121	0.000618	0.046446	-0.35085
LANCL1	LanC like glutathione S-transferase 1	0.829521	0.001651	0.051413	-0.26965
DCAF8	DDB1 and CUL4 associated factor 8	0.829573	0.000436	0.042137	-0.26956
PLS3	plastin 3	0.757469	0.023522	0.095299	-0.40074
SNX33	sorting nexin 33	0.819036	0.009621	0.071965	-0.288
CLASP1	cytoplasmic linker associated protein 1	0.803969	0.01194	0.076865	-0.31479
DNAJB4	DnaJ heat shock protein family (Hsp40) member B4	0.788909	0.00356	0.05435	-0.34207
LOC100345190	ubiquitin-conjugating enzyme E2 L3	0.775673	0.031711	0.106728	-0.36648
SH3BGRL3	SH3 domain binding glutamate rich protein like 3	0.81458	0.049229	0.129113	-0.29587
OXR1	oxidation resistance 1	0.816283	0.004692	0.057495	-0.29286
SH3BGRL	SH3 domain binding glutamate rich protein like	0.797484	0.010021	0.072983	-0.32647
LOC100345691	protein S100-A13	0.76939	0.04168	0.120444	-0.37821
EFEMP1	EGF containing fibulin extracellular matrix protein 1	0.718007	0.017769	0.086129	-0.47793
GSS	glutathione synthetase	0.825928	0.013742	0.080308	-0.27591
P3H4	prolyl 3-hydroxylase family member 4 (inactive)	0.777547	0.005928	0.062795	-0.363
LOC100346112	myosin regulatory light polypeptide 9	0.832366	0.031914	0.1072	-0.26471
LXN	latexin	0.756733	0.007226	0.065388	-0.40214
CIAPIN1	cytokine induced apoptosis inhibitor 1	0.723435	0.009976	0.072983	-0.46707
NEO1	neogenin 1	0.803727	0.006931	0.065034	-0.31522
ANXA6	annexin A6	0.790746	0.007744	0.066928	-0.33871
GPX8	glutathione peroxidase 8 (putative)	0.793423	0.00326	0.053785	-0.33384
NR2F2	nuclear receptor subfamily 2 group F member 2	0.723355	0.000445	0.042137	-0.46722
JPT1	Jupiter microtubule associated homolog 1	0.725417	0.01933	0.088151	-0.46312
ASGR1	asialoglycoprotein receptor 1	0.711352	0.007957	0.067257	-0.49136
AKAP12	A-kinase anchoring protein 12	0.824567	0.000574	0.044402	-0.27829
HIRIP3	HIRA interacting protein 3	0.79964	0.009691	0.072115	-0.32258
PRDX1	peroxiredoxin 1	0.7991	0.008818	0.069448	-0.32355
FNTA	farnesyltransferase, CAAX box, alpha	0.828711	0.012275	0.077666	-0.27106
NAGK	N-acetylglucosamine kinase	0.793423	0.003533	0.05435	-0.33384
FBLN5	fibulin 5	0.776725	0.004207	0.055976	-0.36452
TXNDC17	thioredoxin domain containing 17	0.818733	0.035727	0.11123	-0.28854
COL1A1	collagen type I alpha 1 chain	0.473594	0.006458	0.064546	-1.07828

PAFAH1B2	platelet activating factor acetylhydrolase 1b catalytic subunit 2	0.750073	0.034998	0.110441	-0.4149
PKP2	plakophilin 2	0.492289	1.61E-05	0.027437	-1.02242
NDUFA4	NDUFA4 mitochondrial complex associated	0.826736	0.023652	0.095299	-0.2745
DIAPH2	diaphanous related formin 2	0.832621	0.030761	0.105686	-0.26427
AARS1	alanyl-tRNA synthetase 1	0.779591	0.001224	0.050296	-0.35921
LOC100348562	TRPM8 channel-associated factor 1	0.774623	0.000122	0.037192	-0.36843
STAT3	signal transducer and activator of transcription 3	0.797484	0.015191	0.082389	-0.32647
CSRP2	cysteine and glycine rich protein 2	0.804511	0.011976	0.076865	-0.31382
IGSF3	immunoglobulin superfamily member 3	0.742666	0.002024	0.051413	-0.42921
TMSB10	thymosin beta 10	0.666667	0.035775	0.111312	-0.58496
ZNF706	zinc finger protein 706	0.618123	0.005153	0.058161	-0.69403
TBCB	tubulin folding cofactor B	0.758277	0.020893	0.091552	-0.3992
LZTS2	leucine zipper tumor suppressor 2	0.747742	0.000125	0.037192	-0.41939
LOC100349535	dnaJ homolog subfamily C member 8	0.737833	0.010946	0.075267	-0.43863
PDCD5	programmed cell death 5	0.812387	0.017473	0.085726	-0.29976
ANXA3	annexin A3	0.784652	0.015098	0.082059	-0.34988
ELP3	elongator acetyltransferase complex subunit 3	0.695873	0.043318	0.122136	-0.5231
LOC100349925	small EDRK-rich factor 2	0.683502	0.011316	0.07577	-0.54898
STOM	stomatin	0.785417	0.003091	0.053785	-0.34847
PFN1	profilin 1	0.766559	0.019454	0.088268	-0.38353
NEGR1	neuronal growth regulator 1	0.775673	0.007207	0.065388	-0.36648
SERPINB6	serpin family B member 6	0.737833	0.049581	0.12937	-0.43863
CADM3	cell adhesion molecule 3	0.774327	0.016774	0.084725	-0.36898
TKT	transketolase	0.689189	0.004033	0.055136	-0.53703
LOC100350515	tubulin-specific chaperone A	0.757762	0.049735	0.12937	-0.40018
RSBN1L	round spermatid basic protein 1 like	0.821439	0.001433	0.050986	-0.28377
SEPTIN7	septin 7	0.823959	0.003005	0.053785	-0.27936
UGP2	UDP-glucose pyrophosphorylase 2	0.823458	0.045512	0.123879	-0.28023
PCMTD1	protein-L-isoaspartate (D-aspartate) O-methyltransferase domain containing 1	0.728111	0.012369	0.077666	-0.45777
SEPTIN11	septin 11	0.823154	0.003125	0.053785	-0.28077
DCUN1D1	defective in cullin neddylation 1 domain containing 1	0.818678	0.034034	0.109134	-0.28863
PHLDB2	pleckstrin homology like domain family B member 2	0.815981	0.043839	0.122776	-0.29339
DPYSL2	dihydropyrimidinase like 2	0.794795	0.011036	0.075307	-0.33135
PGM2	phosphoglucomutase 2	0.829826	0.014643	0.081406	-0.26912
DNAJB6	DnaJ heat shock protein family (Hsp40) member B6	0.805597	0.000112	0.037192	-0.31187
GPC4	glypican 4	0.783591	0.009304	0.070949	-0.35183
GPC3	glypican 3	0.778008	0.002571	0.05234	-0.36214
DDAH2	dimethylarginine dimethylaminohydrolase 2	0.762926	0.020158	0.089945	-0.39039
ENO1	enolase 1	0.804269	0.034942	0.110437	-0.31425
HPCA	hippocalcin	0.820941	0.016604	0.084494	-0.28465
FLRT2	fibronectin leucine rich transmembrane protein 2	0.565354	0.010039	0.072983	-0.82277
PDAP1	PDGFA associated protein 1	0.806442	0.004274	0.056138	-0.31036

MOCS3	molybdenum cofactor synthesis 3	0.74216	0.005554	0.060757	-0.4302
PARK7	Parkinsonism associated deglycase	0.823154	0.022382	0.093892	-0.28077
APPL1	adaptor protein, phosphotyrosine interacting with PH domain and leucine zipper 1	0.823458	0.040137	0.118808	-0.28023
RANBP1	RAN binding protein 1	0.815431	0.046305	0.124708	-0.29436
PGD	phosphogluconate dehydrogenase	0.807773	0.031596	0.10666	-0.30798
ZBTB8OS	zinc finger and BTB domain containing 8 opposite strand	0.774394	0.006386	0.06446	-0.36886
COL14A1	collagen type XIV alpha 1 chain	0.642935	0.001031	0.049705	-0.63725
SBDS	SBDS ribosome maturation factor	0.770139	0.04163	0.120444	-0.37681
LOC100353962	alanyl-tRNA editing protein Aarsd1	0.782531	0.026762	0.099943	-0.35378
PRRX1	paired related homeobox 1	0.677383	0.006963	0.065034	-0.56196
TLN2	talin 2	0.804511	0.00129	0.050296	-0.31382
GLO1	glyoxalase I	0.76649	0.016878	0.084725	-0.38366
RBP1	retinol binding protein 1	0.805355	0.04916	0.128997	-0.3123
PDCD4	programmed cell death 4	0.512478	0.001807	0.051413	-0.96444
CLYBL	citramalyl-CoA lyase	0.830131	0.010213	0.073181	-0.26859
SELENBP1	selenium binding protein 1	0.740429	0.011579	0.076329	-0.43357
UHRF2	ubiquitin like with PHD and ring finger domains 2	0.716042	0.000355	0.042137	-0.48188
PIN4	peptidylprolyl cis/trans isomerase, NIMA-interacting 4	0.709402	0.007098	0.065388	-0.49533
PRPS1	phosphoribosyl pyrophosphate synthetase 1	0.777251	0.000472	0.042763	-0.36355
P4HA2	prolyl 4-hydroxylase subunit alpha 2	0.760563	0.002219	0.052154	-0.39486
SDC2	syndecan 2	0.779591	0.018696	0.087113	-0.35921
CSAD	cysteine sulfinic acid decarboxylase	0.793186	0.015096	0.082059	-0.33427
FRY	FRY microtubule binding protein	0.824513	0.004871	0.057873	-0.27839
CFB	complement factor B	0.774098	0.002072	0.051697	-0.36941
ADK	adenosine kinase	0.8226	0.024079	0.096237	-0.28174
LOC100356012	cytochrome P450 4V2	0.740139	0.023952	0.095976	-0.43413
LOC100356307	glutathione S-transferase Mu 1	0.631049	0.037036	0.113506	-0.66417
PFDN5	prefoldin subunit 5	0.820941	0.042018	0.121145	-0.28465
FZD1	frizzled class receptor 1	0.63505	0.007467	0.066045	-0.65506
NCOA6	nuclear receptor coactivator 6	0.812689	0.032313	0.107703	-0.29923
TBX18	T-box transcription factor 18	0.784885	0.000324	0.041472	-0.34945
LIX1L	limb and CNS expressed 1 like	0.617956	0.000294	0.041472	-0.69442
GCHFR	GTP cyclohydrolase I feedback regulator	0.672428	0.001954	0.051413	-0.57255
PRRC2B	proline rich coiled-coil 2B	0.831756	0.00652	0.064807	-0.26577
C1H11orf68	chromosome 1 open reading frame, human C11orf68	0.80072	0.001134	0.050296	-0.32063
PTPN14	protein tyrosine phosphatase non-receptor type 14	0.757984	0.040745	0.119422	-0.39976
ITM2B	integral membrane protein 2B	0.805296	0.01002	0.072983	-0.31241
UBE2E3	ubiquitin conjugating enzyme E2 E3	0.825928	0.011625	0.076537	-0.27591
FHL1	four and a half LIM domains 1	0.740719	0.040839	0.119433	-0.433
BCAM	basal cell adhesion molecule (Lutheran blood group)	0.690423	0.00188	0.051413	-0.53445
EGLN1	egl-9 family hypoxia inducible factor 1	0.783061	0.002243	0.052154	-0.3528
EFNB2	ephrin B2	0.760787	0.008044	0.067257	-0.39444

DBNL	drebrin like	0.824513	0.015522	0.082776	-0.27839
LTA4H	leukotriene A4 hydrolase	0.786246	0.006199	0.063744	-0.34695
PCNP	PEST proteolytic signal containing nuclear protein	0.829573	0.003265	0.053785	-0.26956
RGS10	regulator of G protein signaling 10	0.719484	0.013419	0.079719	-0.47497
SEPTIN10	septin 10	0.766784	0.00866	0.068883	-0.38311
SLC29A1	solute carrier family 29 member 1 (Augustine blood group)	0.739925	0.010326	0.073445	-0.43455
LOC100357917	glutathione S-transferase Mu 1	0.779359	0.045926	0.124194	-0.35964
TCEA3	transcription elongation factor A3	0.79964	0.046981	0.125749	-0.32258
DSTN	destrin, actin depolymerizing factor	0.796707	0.016769	0.084725	-0.32788
C16H1orf198	chromosome 16 open reading frame, human C1orf198	0.733603	0.011312	0.07577	-0.44693
LOC100358177	glutathione S-transferase Mu 1	0.773574	0.046821	0.125635	-0.37039
PCOLCE	procollagen C-endopeptidase enhancer	0.56439	0.002474	0.052154	-0.82524
ITSN1	intersectin 1	0.819284	0.011927	0.076865	-0.28756
PEPD	peptidase D	0.818182	0.020201	0.08996	-0.28951
PFN2	profilin 2	0.776725	3.27E-05	0.037192	-0.36452
LIMCH1	LIM and calponin homology domains 1	0.744186	0.004666	0.057495	-0.42626
LOC100358590	cytochrome P450 1B1	0.811047	0.022055	0.093398	-0.30214
ANKMY2	ankyrin repeat and MYND domain containing 2	0.830384	0.000683	0.04664	-0.26815
LAMB1	laminin subunit beta 1	0.830943	0.010089	0.072983	-0.26718
PTEN	phosphatase and tensin homolog	0.720677	0.024648	0.096878	-0.47258
DNAH9	dynein axonemal heavy chain 9	0.807472	0.036902	0.113232	-0.30852
TLN1	talin 1	0.792413	0.014951	0.082012	-0.33567
CSRP1	cysteine and glycine rich protein 1	0.747016	0.025355	0.097773	-0.42079
HDAC5	histone deacetylase 5	0.815981	0.00977	0.072344	-0.29339
PRPSAP2	phosphoribosyl pyrophosphate synthetase associated protein 2	0.830689	0.001169	0.050296	-0.26762
LOC103344972	protein S100-A4	0.732891	0.049748	0.12937	-0.44833
CCDC25	coiled-coil domain containing 25	0.763668	0.031279	0.106323	-0.38898
PIN1	peptidylprolyl cis/trans isomerase, NIMA-interacting 1	0.827901	0.028051	0.102076	-0.27247
LOC103346005	tensin-3	0.816778	0.002999	0.053785	-0.29198
CYTH3	cytohesin 3	0.817383	0.008083	0.067257	-0.29092
JPT2	Jupiter microtubule associated homolog 2	0.807229	0.028038	0.102076	-0.30895
CDC42BPG	CDC42 binding protein kinase gamma	0.783591	0.002447	0.052154	-0.35183
GFUS	GDP-L-fucose synthase	0.767826	0.006958	0.065034	-0.38115
AGRN	agrin	0.778832	0.001706	0.051413	-0.36062
LOC103347221	nidogen-1	0.770956	0.033479	0.108836	-0.37528
LOC103347362	collagen alpha-1(XVIII) chain-like	0.718007	0.009098	0.070109	-0.47793
LAMA5	laminin subunit alpha 5	0.792949	0.006862	0.065034	-0.3347
LOC103347571	latent-transforming growth factor beta-binding protein 3	0.552795	0.000429	0.042137	-0.85518
LOC103347580	AH receptor-interacting protein	0.831197	0.018191	0.086318	-0.26674
TSPAN8	tetraspanin 8	0.734605	0.003629	0.05435	-0.44496
ASPN	asporin	0.762338	0.000296	0.041472	-0.3915

LOC103349801	cytochrome c oxidase subunit 7A2, mitochondrial	0.818182	0.000221	0.041472	-0.28951
MRC2	mannose receptor C type 2	0.776429	0.00425	0.056057	-0.36507
NR2F1	nuclear receptor subfamily 2 group F member 1	0.745708	0.00028	0.041472	-0.42332
LAMA4	laminin subunit alpha 4	0.801802	0.011788	0.076865	-0.31868

Supplementary Table S4

Proteins up-regulated in dermal papilla cells (DPCs), with 72 hours of TM (5 μM) treatment.

protein	Description	FC	pValue	FDR	log2FC
NES	nestin	1.386635	0.003203	0.053785	0.471588
SLC7A5	solute carrier family 7 member 5	1.500417	0.000515	0.043891	0.585363
JUN	Jun proto-oncogene, AP-1 transcription factor subunit	1.299234	0.002139	0.052154	0.377661
HMOX1	heme oxygenase 1	1.370209	0.001867	0.051413	0.454396
SHMT2	serine hydroxymethyltransferase 2	1.201835	0.002986	0.053785	0.265239
SLC3A2	solute carrier family 3 member 2	1.375446	0.008075	0.067257	0.459899
HMOX2	heme oxygenase 2	1.298353	0.005002	0.058161	0.376683
SOAT1	sterol O-acyltransferase 1	1.218935	0.006361	0.06446	0.285621
PLAT	plasminogen activator, tissue type	1.207506	0.049894	0.129394	0.27203
SERPINE1	serpin family E member 1	1.457002	0.023278	0.094851	0.543003
LOC100125981	solute carrier family 2, facilitated glucose transporter member 3	1.43309	0.000253	0.041472	0.519129
ZNF800	zinc finger protein 800	1.411576	0.000121	0.037192	0.497306
NPM1	nucleophosmin (nucleolar phosphoprotein B23, numatrin)	1.438033	0.000161	0.03912	0.524097
RBM39	RNA binding motif protein 39	1.224694	0.000719	0.04664	0.292421
PVR	PVR cell adhesion molecule	1.242152	9.45E-05	0.037192	0.312842
ATF3	activating transcription factor 3	1.501042	0.000863	0.047924	0.585964
LOC100337853	nucleolar GTP-binding protein 1	1.208318	0.00164	0.051413	0.273
DDX18	DEAD-box helicase 18	1.250188	0.000936	0.048318	0.322145
TOMM7	translocase of outer mitochondrial membrane 7	1.218565	0.031759	0.106746	0.285183
SLC29A3	solute carrier family 29 member 3	1.243829	0.018208	0.086318	0.314789
KIFC1	kinesin family member C1	1.228826	0.003102	0.053785	0.297281
PRCC	proline rich mitotic checkpoint control factor	1.227171	0.002195	0.052154	0.295337
RPS19BP1	ribosomal protein S19 binding protein 1	1.357564	0.008076	0.067257	0.44102
PIGO	phosphatidylinositol glycan anchor biosynthesis class O	1.203085	0.017297	0.085295	0.266738
KIF20B	kinesin family member 20B	1.328677	0.001769	0.051413	0.40999
EMILIN2	elastin microfibril interfacer 2	1.208318	0.024626	0.096878	0.273
NFKB2	nuclear factor kappa B subunit 2	1.20301	0.001075	0.050296	0.266649
LIPM	lipase family member M	1.300115	0.002288	0.052154	0.378639
ARMC10	armadillo repeat containing 10	1.271385	0.042475	0.121478	0.346401
NOM1	nucleolar protein with MIF4G domain 1	1.253944	0.005737	0.061767	0.326473
BARD1	BRCA1 associated RING domain 1	1.312139	0.002033	0.051413	0.39192
RBM33	RNA binding motif protein 33	1.205882	0.04309	0.121953	0.270089
MAFF	MAF bZIP transcription factor F	1.910723	0.000434	0.042137	0.934119
DDX21	DExD-box helicase 21	1.263774	0.00149	0.050986	0.337738
ING5	inhibitor of growth family member 5	1.380952	0.019168	0.087907	0.465664
LYAR	Ly1 antibody reactive	1.22387	0.002539	0.052306	0.29145
LOC100342069	interferon-induced protein with tetratricopeptide repeats 1B	1.454583	0.027425	0.101237	0.540605
TMEM231	transmembrane protein 231	1.242991	0.014538	0.081167	0.313815
C4H12orf73	chromosome 4 C12orf73 homolog	1.369128	0.002514	0.052177	0.453257
HNRNPA2B1	heterogeneous nuclear ribonucleoprotein A2/B1	1.212758	0.001815	0.051413	0.278292

NIP7	nucleolar pre-rRNA processing protein NIP7	1.220126	0.000199	0.041472	0.28703
MKI67	marker of proliferation Ki-67	1.308077	0.000405	0.042137	0.387447
AIFM2	apoptosis inducing factor mitochondria associated 2	1.219756	0.004287	0.05616	0.286592
MCM10	minichromosome maintenance 10 replication initiation factor	1.268053	0.016525	0.084479	0.342615
CENPF	centromere protein F	1.263297	0.004169	0.055756	0.337194
IRF9	interferon regulatory factor 9	1.206694	0.010128	0.072983	0.271059
ZKSCAN1	zinc finger with KRAB and SCAN domains 1	1.321981	0.011429	0.076079	0.402702
TM7SF3	transmembrane 7 superfamily member 3	1.245135	0.00511	0.058161	0.316302
SLC30A6	solute carrier family 30 member 6	1.208763	0.0046	0.057495	0.273531
KIF20A	kinesin family member 20A	1.304531	0.002429	0.052154	0.383532
FAM111A	FAM111 trypsin like peptidase A	1.330485	0.020347	0.090201	0.411953
CKAP2	cytoskeleton associated protein 2	1.586207	0.01289	0.078493	0.665581
KIF11	kinesin family member 11	1.414487	0.004613	0.057495	0.500279
LOC100346247	MKI67 FHA domain-interacting nucleolar phosphoprotein	1.213574	0.001973	0.051413	0.279262
MBLAC2	metallo-beta-lactamase domain containing 2	1.286095	0.000245	0.041472	0.362997
HSD17B12	hydroxysteroid 17-beta dehydrogenase 12	1.333722	0.001086	0.050296	0.415458
LYSMD3	LysM domain containing 3	1.302379	0.00616	0.063744	0.381149
MYEF2	myelin expression factor 2	1.201468	0.000445	0.042137	0.264798
UTP14A	UTP14A small subunit processome component	1.226716	0.010692	0.074537	0.294801
INO80E	INO80 complex subunit E	1.238806	0.000424	0.042137	0.30895
RCL1	RNA terminal phosphate cyclase like 1	1.206694	0.009394	0.071418	0.271059
CDCA3	cell division cycle associated 3	1.332815	0.000402	0.042137	0.414476
POLR2H	RNA polymerase II, I and III subunit H	1.214022	0.006134	0.063744	0.279795
SLF2	SMC5-SMC6 complex localization factor 2	1.288821	0.017907	0.086276	0.366052
SLC1A4	solute carrier family 1 member 4	1.32378	0.001434	0.050986	0.404663
LOC100349005	guanylate-binding protein 4	1.207506	0.027578	0.101584	0.27203
PSPH	phosphoserine phosphatase	1.233346	0.006776	0.064844	0.302577
MTHFD2	methylenetetrahydrofolate dehydrogenase (NADP+ dependent) 2, methenyltetrahydrofolate cyclohydrolase	1.784687	0.000833	0.047824	0.835671
NCKAP5	NCK associated protein 5	1.206694	0.003837	0.054946	0.271059
DDX43	DEAD-box helicase 43	1.325194	0.003111	0.053785	0.406203
SHCBP1	SHC binding and spindle associated 1	1.306421	0.003686	0.05475	0.385619
TOPBP1	DNA topoisomerase II binding protein 1	1.231313	0.030516	0.105129	0.300197
DLAT	dihydrolipoamide S-acetyltransferase	1.226345	0.004405	0.056664	0.294365
LOC100349257	guanylate-binding protein 5	1.643172	0.008176	0.067653	0.716483
MYBBP1A	MYB binding protein 1a	1.231313	0.002831	0.053261	0.300197
TOP1	DNA topoisomerase I	1.201468	0.01831	0.086375	0.264798
NUSAP1	nucleolar and spindle associated protein 1	1.231313	0.009756	0.07234	0.300197
PPP1R10	protein phosphatase 1 regulatory subunit 10	1.264151	0.000538	0.044402	0.338169
SRSF4	serine and arginine rich splicing factor 4	1.215657	0.006469	0.064546	0.281737
ZFX	zinc finger protein X-linked	1.225519	0.009519	0.071732	0.293393
RBBP6	RB binding protein 6, ubiquitin ligase	1.203452	0.001207	0.050296	0.267179
NUDT19	nudix hydrolase 19	1.357957	0.002331	0.052154	0.441438
RETREG3	reticulophagy regulator family member 3	1.232143	0.021107	0.091932	0.30117

CNNM4	cyclin and CBS domain divalent metal cation transport mediator 4	1.324167	0.003019	0.053785	0.405085
ANLN	anillin actin binding protein	1.289203	0.031411	0.106488	0.366479
DDX5	DEAD-box helicase 5	1.234637	6.89E-05	0.037192	0.304087
NOP14	NOP14 nucleolar protein	1.255263	0.002014	0.051413	0.32799
NPM3	nucleophosmin/nucleoplasmin 3	1.282725	0.030149	0.105064	0.359211
VMP1	vacuole membrane protein 1	1.233346	0.006627	0.064844	0.302577
DGCR8	DGCR8 microprocessor complex subunit	1.268431	0.002074	0.051697	0.343045
SNU13	small nuclear ribonucleoprotein 13	1.208686	0.003123	0.053785	0.27344
INCENP	inner centromere protein	1.51995	0.005332	0.059217	0.604023
ALDH5A1	aldehyde dehydrogenase 5 family member A1	1.216476	0.001715	0.051413	0.282708
KPNA2	karyopherin subunit alpha 2	1.259887	0.003836	0.054946	0.333294
MRS2	magnesium transporter MRS2	1.24635	0.008601	0.068883	0.317709
FUS	FUS RNA binding protein	1.219675	0.003911	0.055136	0.286496
SDC4	syndecan 4	1.361811	0.007034	0.065388	0.445527
LOC100352568	soluble calcium-activated nucleotidase 1	1.210313	0.006976	0.065034	0.27538
KLHDC4	kelch domain containing 4	1.413516	0.011121	0.075307	0.499288
RBM34	RNA binding motif protein 34	1.230026	0.000809	0.047824	0.298689
SDAD1	SDA1 domain containing 1	1.206326	0.009488	0.071601	0.27062
ZC3H11A	zinc finger CCCH-type containing 11A	1.277525	0.001385	0.050986	0.353351
SLC25A25	solute carrier family 25 member 25	1.303263	0.001142	0.050296	0.382128
CDCA5	cell division cycle associated 5	1.633172	0.043094	0.121953	0.707677
SDHAF2	succinate dehydrogenase complex assembly factor 2	1.209945	0.017594	0.085874	0.274941
NOLC1	nucleolar and coiled-body phosphoprotein 1	1.333722	0.001402	0.050986	0.415458
ISG15	ISG15 ubiquitin like modifier	1.275797	0.018928	0.08783	0.351398
LOC100355052	trace amine-associated receptor 8-like	1.220577	0.008132	0.067556	0.287564
DNAJB2	DnaJ heat shock protein family (Hsp40) member B2	1.352549	0.000281	0.041472	0.435681
LOC100355345	chromosome unknown open reading frame, human C1orf35	1.30188	0.006365	0.06446	0.380596
RRP7A	ribosomal RNA processing 7 homolog A	1.259036	4.33E-05	0.037192	0.33232
CLDND1	claudin domain containing 1	1.20066	0.000799	0.047824	0.263828
SMTN	smoothelin	1.420734	0.013199	0.078959	0.506637
ATAD2	ATPase family AAA domain containing 2	1.222222	0.003594	0.05435	0.289507
AURKB	aurora kinase B	1.394254	0.012309	0.077666	0.479493
KRR1	KRR1 small subunit processome component homolog	1.240105	0.006173	0.063744	0.310462
NOL8	nucleolar protein 8	1.280502	0.000968	0.048502	0.356709
C4A	complement C4A (Rodgers blood group)	1.244203	0.042505	0.121478	0.315222
YIPF3	Yip1 domain family member 3	1.260739	0.002337	0.052154	0.334269
UTP3	UTP3 small subunit processome component	1.208318	0.008163	0.067653	0.273
CREB5	cAMP responsive element binding protein 5	1.217295	0.013263	0.079068	0.283679
TOP2A	DNA topoisomerase II alpha	1.496256	0.00072	0.04664	0.581357
C16H1orf131	chromosome 16 open reading frame, human C1orf131	1.318393	6.28E-06	0.01604	0.39878
LOC100357965	zinc finger protein 271-like	1.301496	0.035336	0.110891	0.380171
BYSL	bystin like	1.238806	0.01099	0.075307	0.30895
DNAJB12	DnaJ heat shock protein family (Hsp40) member B12	1.257336	0.001987	0.051413	0.330371

LOC100358539	guanylate-binding protein 1	1.279635	0.033135	0.10849	0.355733
SPAG1	sperm associated antigen 1	1.300613	0.001371	0.050986	0.379192
NOP2	NOP2 nucleolar protein	1.29709	0.000425	0.042137	0.375279
TOX4	TOX high mobility group box family member 4	1.238806	0.000687	0.04664	0.30895
LOC100358742	histone H2AX	1.205439	0.009677	0.072115	0.269559
PRKN	parkin RBR E3 ubiquitin protein ligase	1.316602	0.015702	0.083192	0.39682
FAM162A	family with sequence similarity 162 member A	1.220577	0.001653	0.051413	0.287564
TTK	TTK protein kinase	1.286204	0.012313	0.077666	0.36312
MCL1	MCL1 apoptosis regulator, BCL2 family member	1.213205	0.010454	0.073635	0.278824
RHOF	ras homolog family member F, filopodia associated	1.250938	0.039984	0.118628	0.32301
RELB	RELB proto-oncogene, NF-kB subunit	1.200587	0.012847	0.078417	0.26374
PC	pyruvate carboxylase	1.244203	0.000321	0.041472	0.315222
NOP53	NOP53 ribosome biogenesis factor	1.279635	0.000507	0.043891	0.355733
BHLHE41	basic helix-loop-helix family member e41	1.217295	0.005216	0.058189	0.283679
TEX264	testis expressed 264, ER-phagy receptor	1.301496	0.003995	0.055136	0.380171
C9H18orf32	chromosome 9 open reading frame, human C18orf32	1.325068	0.002316	0.052154	0.406066
GNG4	G protein subunit gamma 4	1.342311	0.004154	0.055702	0.424719
SLC30A1	solute carrier family 30 member 1	1.290076	0.001991	0.051413	0.367456
CHTOP	chromatin target of PRMT1	1.273967	0.003176	0.053785	0.349328
